# Delivery of engineered extracellular vesicles with miR-29b editing system for muscle atrophy therapy

**DOI:** 10.1186/s12951-022-01508-4

**Published:** 2022-06-27

**Authors:** Rui Chen, Weilin Yuan, Yongjun Zheng, Xiaolan Zhu, Bing Jin, Tingting Yang, Yuwei Yan, Wanru Xu, Hongjian Chen, Juan Gao, Guoping Li, Priyanka Gokulnath, Gururaja Vulugundam, Jin Li, Junjie Xiao

**Affiliations:** 1 Institute of Geriatrics, The Sixth People’s Hospital of Nantong), School of Medicine, Shanghai University, Affiliated Nantong Hospital of Shanghai University, Shanghai University, Nantong, 226011 China; 2 Cardiac Regeneration and Ageing Lab, Institute of Cardiovascular Sciences, Shanghai Engineering Research Center of Organ Repair, School of Life Science, Shanghai University, 333 Nan Chen Road, Shanghai, 200444 China; 3 Division of Pain Management, Huadong Hospital Affiliated to Fudan University, Shanghai, 200040 China; 4 Cardiovascular Division of the Massachusetts General Hospital, Harvard Medical School, Boston, MA 02114 USA; 5 Institute of Biochemistry and Cellular Biology, National Research Council of Italy, Napoli, 80131 Italy

**Keywords:** Engineered extracellular vesicles, miR-29b, CRISPR/Cas9, Muscle atrophy, Therapy

## Abstract

Muscle atrophy is a frequently observed complication, characterized by the loss of muscle mass and strength, which diminishes the quality of life and survival. No effective therapy except exercise is currently available. In our previous study, repressing miR-29b has been shown to reduce muscle atrophy. In our current study, we have constructed artificially engineered extracellular vesicles for the delivery of CRISPR/Cas9 to target miR-29b (EVs-Cas9-29b). EVs-Cas9-29b has shown a favorable functional effect with respect to miR-29b repression in a specific and rapid manner by gene editing. In in vitro conditions, EVs-Cas9-29b could protect against muscle atrophy induced by dexamethasone (Dex), angiotensin II (AngII), and tumor necrosis factor-alpha (TNF-α). And EVs-Cas9-29b introduced in vivo preserved muscle function in the well-established immobilization and denervation-induced muscle atrophy mice model. Our work demonstrates an engineered extracellular vesicles delivery of the miR-29b editing system, which could be potentially used for muscle atrophy therapy.

**Supplementary Information:**

The online version contains supplementary material available at 10.1186/s12951-022-01508-4.

## Introduction

Muscle atrophy is a systemic response to a variety of pathologies such as heart failure, cancer, chronic kidney disease, and chronic obstructive pulmonary disease (COPD). It is also a condition observed as a consequence of denervation, inactivity, and aging. Muscle atrophy is caused by abnormal activation of protein degradation and decreased protein synthesis [1, 2]. Moreover, muscle atrophy is always associated with higher morbidity and mortality as well as a poor prognosis for patients with a low quality of life [3]. Until now, there have been no effective treatments for muscle atrophy except for physical exercise [4–6]. Thus, the development of newer methods to combat this debilitating ailment will benefit a variety of clinical conditions.

miR-29b has been found to be upregulated in a diverse set of diseases with muscle atrophy, such as those induced by denervation, dexamethasone, fasting, cancer cachexia, aging, and heart failure and consequently reducing miR-29b has been observed to attenuate it [4, 7]. Moreover, we also identified that exercise attenuated muscle atrophy by suppressing miR-29b expression [8]. Thus, the inhibition of miR-29b is suggested to be a therapeutic target to counteract muscle atrophy occurring under different disease conditions. Currently, the miRNA inhibitors used commercially by the pharmaceutical industry are designed based on Watson-Crick base pairing. These miRNA inhibitors, including chemically synthesized antagomirs, antisense inhibitors, and locked nucleic acids, are widely used in the pre-clinical stage and in clinical trials [9]. However, the toxicity and unpredictable side effects of these synthesized inhibitors limit the development of anti-miRNA/miRNA mimics drugs. Fortunately, the revolutionary clustered regularly interspaced short palindromic repeat (CRISPR)-associated protein (Cas9) can substitute the miRNA inhibitors as an effective tool for disrupting miRNA.

Furthermore, our former study established CRISPR/Cas9-based editing systems targeting miR-29b to treat muscle atrophy by being packaged into lentivirus and adeno-associated viruses (AAV) to facilitate gene delivery. This gene-editing system has indeed demonstrated a therapeutic effect on multiple types of muscle atrophy by using in vivo mice model [10]. Thus, the miR-29b editing system has been established as a promising method to treat muscle atrophy. However, further studies are required to develop a more appropriate and safer in vivo delivery system to better carry out clinical translational research.

Extracellular vesicles (EVs) are heterogeneous populations of membrane-bound vesicles released by various types of cells [11]. EVs play vital roles in intercellular crosstalk by transferring RNAs, proteins, and lipids from donor cells to recipient cells. The bilayered lipid membrane could protect EV cargo from degradation in the circulation system [12]. Recently, EVs have been explored as vehicles to deliver Cas9 ribonucleoprotein (RNP) [13–20]. Engineered EVs could specifically deliver gene-editing system to the target organs. Because of their low toxicity, low immunogenicity, and high loading capacity [21–24], EVs offer several advantages for delivering CRISPR/Cas9 compared to other systems. In addition, due to the lack of viral nucleic acid genomes, EVs could act as safe and stable delivery vehicles.

In this study, we constructed an artificially engineered extracellular vesicle-based delivery system with CRISPR/Cas9 for targeting miR-29b (EVs-Cas9-29b). EVs-Cas9-29b, through CRISPR/Cas9 gene editing, could disrupt the expression of miR-29b both specifically and rapidly. EVs-Cas9-29b was observed to protect against muscle atrophy in vitro induced by dexamethasone, angiotensin II, and tumor necrosis factor-alpha. Further, EVs-Cas9-29b significantly improved immobilization and denervation-induced muscle atrophy in mice. From these preliminary observations, EVs-Cas9-29b shows promising role in acting as a therapeutic tool for muscle atrophy.

## Results

### Construction of engineered extracellular vesicle-based CRISPR/Cas9 system for disrupting miR-29b

**Fig. 1:**
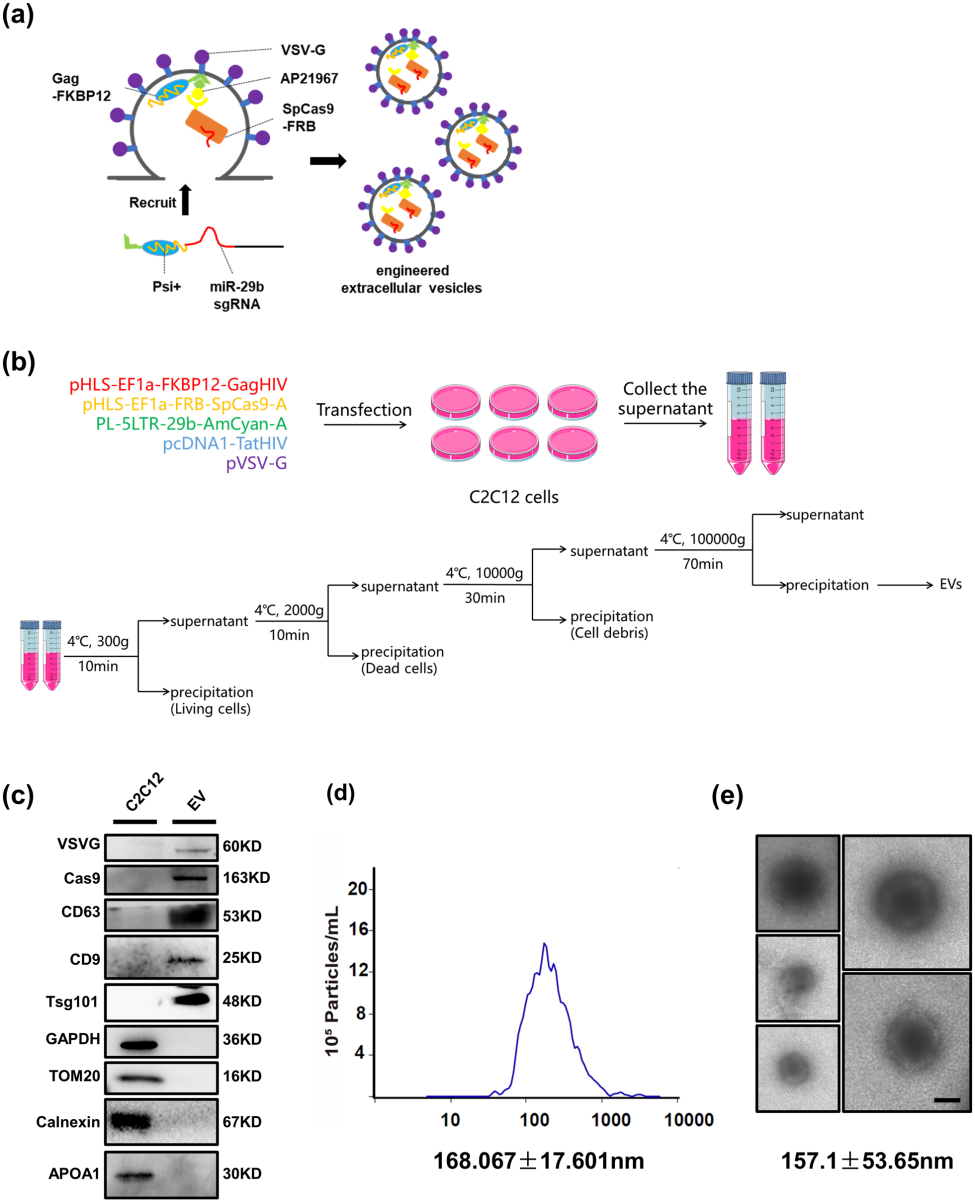
Design of engineered extracellular vesicles delivering miR-29b editing system. **a** Schematic diagram of engineered extracellular vesicles. **b** The flow chart used to synthesize engineered extracellular vesicles. **c** Western blot analysis of Cas9, V-SVG, CD63, CD9, TSG101, GAPDH, TOM20, Calnexin and APOA1 protein in engineered extracellular vesicles and C2C12 cells. **d** Fluorescent nanoparticle tracking analysis (FNTA) to determine the size distribution of engineered extracellular vesicles. **e** Transmission electron microscopic (TEM) analysis of purified engineered extracellular vesicles’ structure and size (scale bar: 100 nm)

We had previously reported a gene-editing system based on CRISPR/Cas9 that could treat muscle atrophy by suppressing the expression of miR-29b [10]. The CRISPR/Cas9 gene-editing system delivered by adeno-associated virus (AAV) or lentivirus could protect against muscle atrophy. Still, the therapeutic efficacy of these systems in established muscle atrophy models was not up to our expectations. The lower efficacy could be due to the delayed expression of viral-vector carrying DNA. In this study, we introduced an engineered extracellular vesicle-based CRISPR/Cas9 system to disrupt the expression of miR-29b to improve both the delivery efficiency and therapeutic efficacy. According to NanoMEDIC (nanomembrane-derived extracellular vesicles for the delivery of macromolecular cargo), a strategy proposed by Akitsu Hotta [14], we constructed an engineered extracellular vesicle to package and deliver the Cas9 protein and a small guide RNA (sgRNA) of miR-29b to the target tissue for its rapid initiation of gene editing as shown in Fig. 1a. Firstly, vesicular stomatitis virus-glycoprotein (VSV-G) is an envelope glycoprotein derived from vesicular stomatitis virus and has been used to modify EVs to increase the efficiency of their invasion of target tissues. Secondly, A/C heterodimerizer (AP21967) is used as a compound ligand to induce EV packaging to recruit Gag proteins of HIV-1 (HIV Gag) and streptococcus pyogenes Cas9 protein (spCas9). As a result, FKBP-binding domain (FRB) N-terminal fused spCas9 and FK506-binding protein (FKBP12)-Gag could be non-covalently linked by AP21967 as an adaptor and recruited into EVs. Thirdly, to recruit miR-29b sgRNA into EVs, we used the guide RNA as shown in our previous study [10]. Further, two lentiviral vector components, Tat activation response element (TAR, trans-activation response element) in the 5′ long terminal repeat (5′ LTR) promoter region, and an extended Psi (Ψ+) packaging signal were included. The Psi (Ψ+) signal binds specifically to the nucleocapsid of Gag and carries sgRNA into EVs, where the sgRNA fragment is cleaved and released by hammerhead (HH) and hepatitis delta virus (HDV) ribozymes.

C2C12 cells were used to produce the EVs, and centrifugation at different speeds was performed to remove other components apart from EVs. The process of EVs production is shown in Fig. 1b. Engineered EVs were characterized by western blotting to assess EV proteins, fluorescent nanoparticle tracking analysis (FNTA), and transmission electron microscope (TEM) assays. Western blot analysis of the engineered EVs revealed that they contain the EV markers such as CD9, CD63, and TSG101, as well as the expected cargo proteins such as modified Cas9, and V-SVG proteins. These engineered EVs are negative for GAPDH, TOM20, Calnexin and APOA1 proteins (Fig. 1c). The size distribution of EVs was confirmed to be 168.067 ± 17.601 nm via FNTA (Fig. 1d). TEM imaging confirmed the size and the membrane-like morphological structure of EVs (Fig. 1e).

**Fig. 2:**
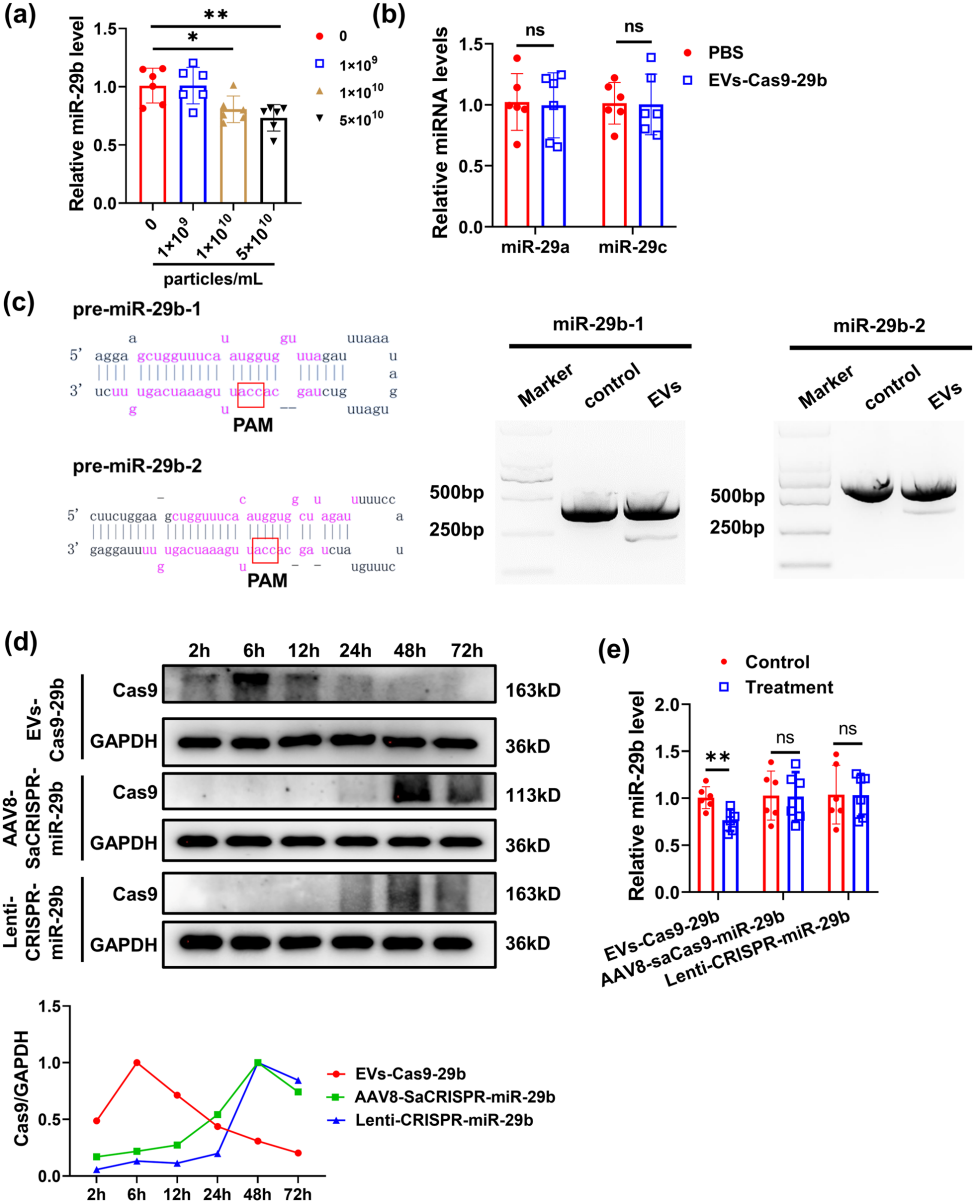
EVs-Cas9-miR-29b inhibits the expression of miR-29b by gene editing. **a** qRT-PCR analysis of the expression of miR-29b in C2C12 myotubes after treatment with EVs-Cas9-29b at different doses (n = 6 per group). **b** qRT-PCR analysis of the expression of miR-29a and miR-29c in C2C12 myotubes after treatment with EVs-Cas9-29b at a dose of 1 × 10^10^ particles/mL (n = 6 per group). **c** Diagram of miR-29b pre-miRNA hairpin structure and the protospacer adjacent motif (PAM) for the gRNA. And T7EI assays for the mutation at the miR-29b-1 and miR-29b-2 in C2C12 myotubes treated with EVs-Cas9-29b. **d** Western blot analysis for the Cas9 protein in C2C12 myotubes after treatment with EVs-Cas9-29b, AAV8-SaCRISPR-miR-29b and Lenti-CRISPR-miR-29b. **e** qRT-qPCR analysis of the expression of miR-29b in C2C12 myotubes after treatment with EVs-Cas9-29b, AAV8-SaCRISPR-miR-29b and Lenti-CRISPR-miR-29b for 24 h (n = 6 per group). Data were presented as mean ± SD. Statistical significance was determined using Student *t* test (a,b,e). *P < 0.05; **P < 0.01; ns, no significance

We next assessed the function of EVs-Cas9-29b. The miR-29b expression levels were analyzed by qRT-PCR, and miR-29b-3p (also known as miR-29b) was observed to be reduced in EVs-Cas9-29b-treated C2C12 myotubes, while the expression of miR-29a and miR-29c was not affected (Fig. 2a, b). Moreover, EVs-Cas9-29b was observed to not affect the expression of miR-29b-5p-1 and miR-29b-5p-2 in C2C12 myotubes (Additional file 1: Fig. S1). The T7 endonuclease 1 (T7E1) mismatch detection assay showed that EVs-Cas9-29b caused significant mutagenesis in the genomic DNA of miR-29b (Fig. 2c). Thus, EVs-Cas9-29b could efficiently disrupt the expression of miR-29b through gene editing. We then wanted to compare the time taken for EVs-Cas9-29b functionality and the viral delivery RNPs (AAV8-SaCRISPR-miR-29b and Lenti-CRISPR-miR-29b) that we used earlier [10]. The viral nucleic acid genome was examined by analyzing the EVs by semi-quantitative PCR and then examining the DNA using agarose gel electrophoresis. And we found that EVs-Cas9-29b lacked any viral DNA (Additional file 1: Fig. S2).

We added EVs-Cas9-29b, AAV8-SaCRISPR-miR-29b and Lenti-CRISPR-miR-29b to C2C12 myotubes, and collected these cells at different time points to analyze the Cas9 protein expression. The Cas9 protein level was highest at 6 h after EVs-Cas9-29b delivery and decreased quickly, whereas after AAV8-SaCRISPR-miR-29b and Lenti-CRISPR-miR-29b treatment, the Cas9 protein was shown to increase at 48 h (Fig. 2d). Furthermore, qRT-PCR on the treated cells showed that EVs-Cas9-29b could silence miR-29b in 24 h, while AAV8-saCRISPR-miR-29b and Lenti-CRISPR-miR-29b did not affect the expression of miR-29b at this short time (Fig. 2e). The rapid increase of Cas9 protein and the corresponding silencing of miR-29b in a short time suggest that the EVs-Cas9-29b delivery system could function as a high-efficiency disruption system against miR-29b and contribute towards muscle atrophy therapy.

**Fig. 3:**
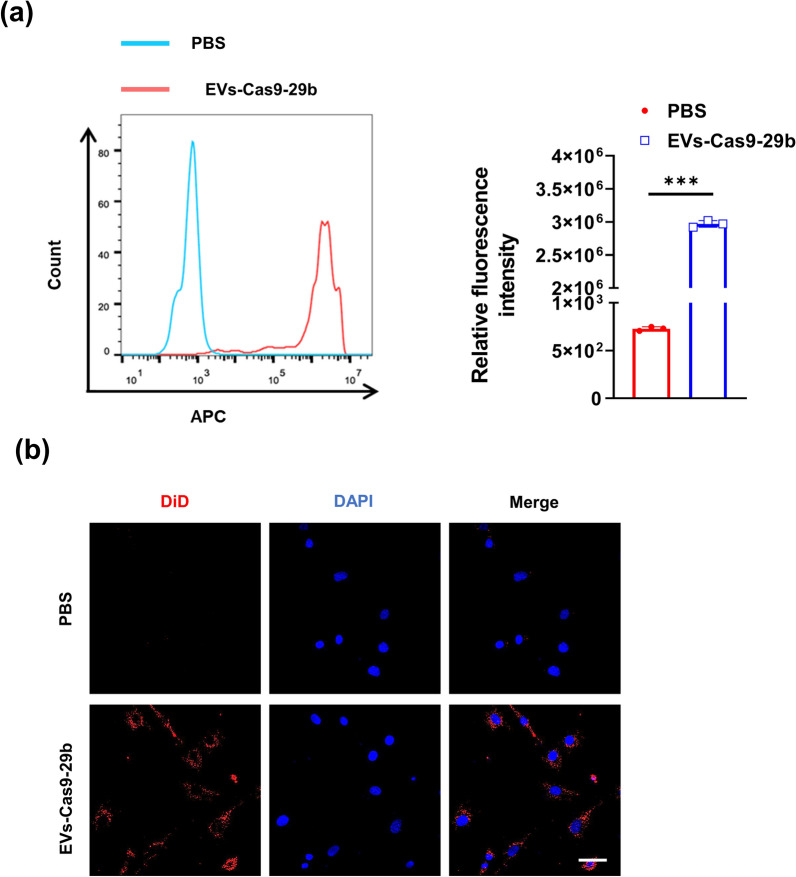
EVs-Cas9-29b uptake in C2C12 cells. **a** The fluorescence intensity of C2C12 cells incubated with DiD-labeled EVs-Cas9-29b for 4 h shown through flow cytometry (n = 3 per group). **b** Representative images of C2C12 cells incubated with DiD-labeled EVs-Cas9-29b for 4 h (n = 3 per group; scale bar: 50 μm), Red, DiD-labeled EV; Blue, DAPI. Data were presented as mean ± SD. Statistical significance was determined using Student t test (**a**). ***P < 0.01

We wanted to confirm the efficiency of EVs-Cas9-29b is primarily due to its uptake by C2C12 cells. Therefore, C2C12 cells incubated with DiD-labeled EVs-Cas9-29b were analyzed using flow cytometry and fluorescence microscopy. Nearly all cells showed EVs-Cas9-29b uptake demonstrated by a strong perinuclear fluorescence (Fig. 3). These results indicated that EVs-Cas9-29b treatment was extremely effective as seen by its high uptake in C2C12 cells.

### **EVs-Cas9-29b attenuates muscle atrophy*in vitro***

**Fig. 4:**
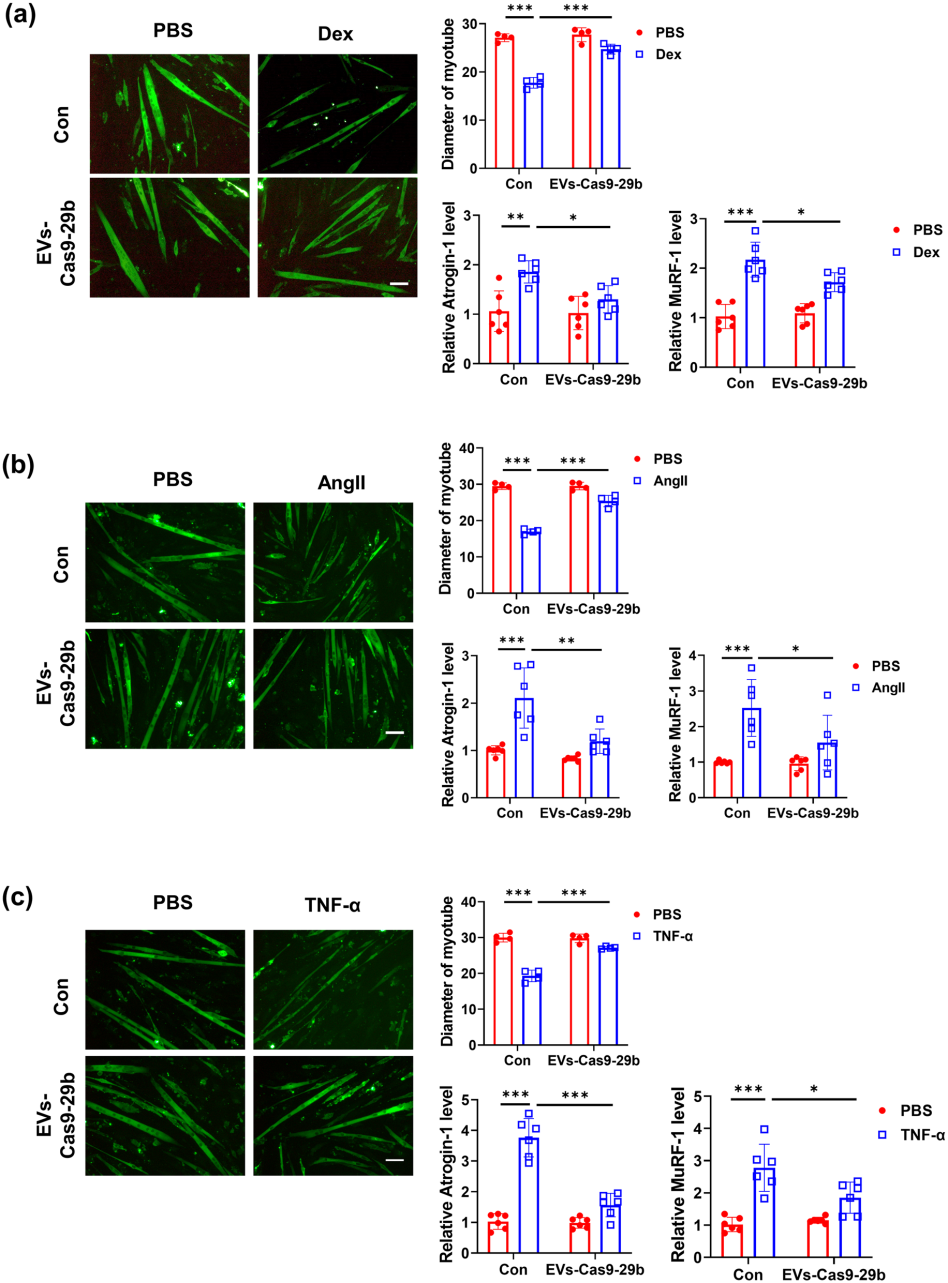
EVs-Cas9-29b attenuates muscle atrophy in vitro. **a** Immunofluorescent staining and quantification (n = 4 per group; scale bar: 100 μm; Green, MF-20, represents the myotube); qRT-PCR analysis of Atrogin-1 and MuRF-1 expression (n = 6 per group) in C2C12 myotubes after treatment with EVs-Cas9-29b in Dex-induced muscle-atrophy model. **b** Immunofluorescent staining and quantification (n = 4 per group; scale bar: 100 μm; Green, MF-20, represents the myotube); qRT-PCR analysis of Atrogin-1 and MuRF-1 expression (n = 6 per group) in C2C12 myotubes after treatment with EVs-Cas9-29b in Ang II-induced muscle-atrophy model. **c** Immunofluorescent staining and quantification (n = 4 per group; scale bar: 100 μm; Green, MF-20, represents the myotube); qRT-PCR analysis of Atrogin-1 and MuRF-1 expression (n = 6 per group) in C2C12 myotubes after treatment with EVs-Cas9-29b in TNF-α-induced muscle-atrophy model. Data were presented as Mean ± SD. Statistical significance was determined by Two-way ANOVA with Tukey test (a-c). *P < 0.05; **P < 0.01 and ***P < 0.001

To understand if EVs-Cas9-29b has any protective effects against atrophy induced in cultured C2C12 myotubes, EVs-Cas9-29b was added to the medium of C2C12 myotubes, and a muscle atrophy model was induced by treatment with dexamethasone (Dex). In Dex-induced muscle atrophy in vitro, EVs-Cas9-29b could reverse the decrease of myotube size and inhibit the elevation of muscle atrophy marker genes such as muscle-specific RING-finger 1 (MuRF-1) and Atrogin-1 (Fig. 4a). Consistently, angiotensin II (Ang II) and tumor necrosis factor alpha (TNF-α)-induced myotube atrophy were also significantly reversed by EVs-Cas9-29b application as determined by the myotube size and expression levels of Atrogin-1 and MuRF-1 (Fig. 4b, c). These data suggest that EVs-Cas9-29b could reverse muscle atrophy in vitro.

### Therapeutic use of EVs-Cas9-29b attenuates immobilization-induced muscle atrophy in vivo

**Fig. 5:**
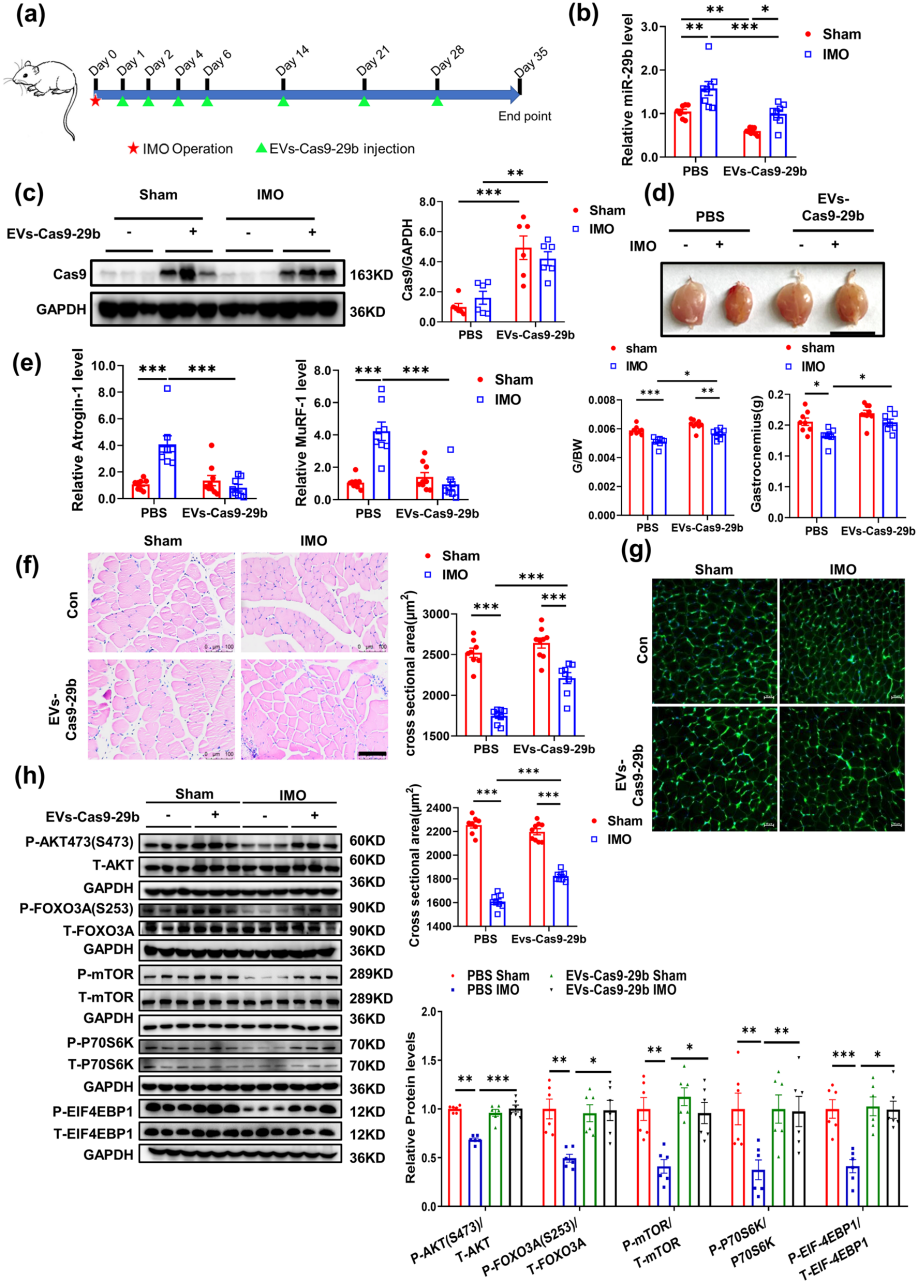
EVs-Cas9-29b therapy attenuate immobilization induced muscle atrophy in vivo. **a** Schematic diagram of experimental design process. **b** qRT-PCR analysis for the expression of miR-29b in gastrocnemius muscle of mice injected with EVs-Cas9-29b in IMO-induced muscle atrophy (n = 8,8,9,8). **c** Western blot analysis for Cas9 protein in gastrocnemius muscle of mice injected with EVs-Cas9-29b in IMO-induced muscle atrophy (n = 6). **d** Gastrocnemius muscle morphology, weight and Gastrocnemius weight/body Weight ratio of mice injected with EVs-Cas9-29b in IMO-induced muscle atrophy (n = 8,8,9,8). **e** qRT-PCR analysis for the expression of Atrogin-1 and MuRF-1 in gastrocnemius muscle of mice injected with EVs-Cas9-29b in IMO-induced muscle atrophy (n = 8,8,9,8). **f–g** HE staining and WGA staining for myofiber of mice injected with EVs-Cas9-29b in IMO-induced muscle atrophy (n = 8,8,9,8; scale bar:100 μm:). **h** Western blot analysis of AKT/FOXO3A/mTOR pathway (AKT, FOXO3A, mTOR, P70S6K, 4EBP1) in gastrocnemius muscle of mice injected with EVs-Cas9-29b in IMO-induced muscle atrophy (n = 6 per group). Data were presented as mean ± SD. Statistical significance was determined by Two-way ANOVA with Tukey test (b-h). *P < 0.05; **P < 0.01 and ***P < 0.001

To investigate the clinical prospects of EVs-Cas9-29b in muscle atrophy, we then determined the therapeutic effects of EVs-Cas9-29b on established muscle atrophy models induced by immobilization (IMO). EVs-Cas9-29b was injected into the gastrocnemius (GA) muscles followed by IMO operation of the hind limbs, and muscle atrophy was evaluated after EV treatment for 5 weeks (Fig. 5a). EVs-Cas9-29b could inhibit the expression of miR-29b in both sham and IMO-treated mice (Fig. 5b). In addition, Cas9 protein was observed to be enriched specifically in the muscle tissues of mice treated with EVs-Cas9-29b, but not in other organs (Fig. 5c and Additional file 1: Fig. S3a). These data indicate that EVs-Cas9-29b was successfully delivered into the skeletal muscle cells and worked effectively, without any side effects in other organs.

The extent of muscle atrophy was then assessed. Compared to the controls, EVs-Cas9-29b therapy significantly increased the weight of the mouse gastrocnemius muscle, repressed the expression of Atrogin-1 and MuRF-1, increased the cross-sectional area of myofibers in IMO-treated mice (Fig. 5d–g). Next, we determined the AKT/FOXO3A/mTOR growth pathway by western blot. In IMO-treated mice, the phosphorylation of AKT(S473), FOXO3A(S253), mTOR, P70S6K, and EIF-4EBP1 were decreased, while EVs-Cas9-29b therapy could reverse that (Fig. 5 h). In addition, Insulin growth factor 1 (IGF1) and phosphatidylinositol 3-kinase (PI3K)(p85α) have been previously identified as target genes of miR-29b [25]. Therefore, we analyzed their protein levels in IMO-treated mice that underwent EVs-Cas9-29b therapy. Western blot showed that EVs-Cas9-29b could increase the IGF-1 and PI3K(p85α) in IMO-treated mice (Additional file 1: Fig. S3b). Besides, EVs-Cas9-29b therapy did not affect inflammation in immobilization-induced muscle atrophy (Additional file 1: Fig. S3c). Taken together, our data suggest that the administration of EVs-Cas9-29b could attenuate muscle atrophy induced by IMO.

### Therapeutic use of EVs-Cas9-29b attenuates denervation-induced muscle atrophy in vivo

**Fig. 6:**
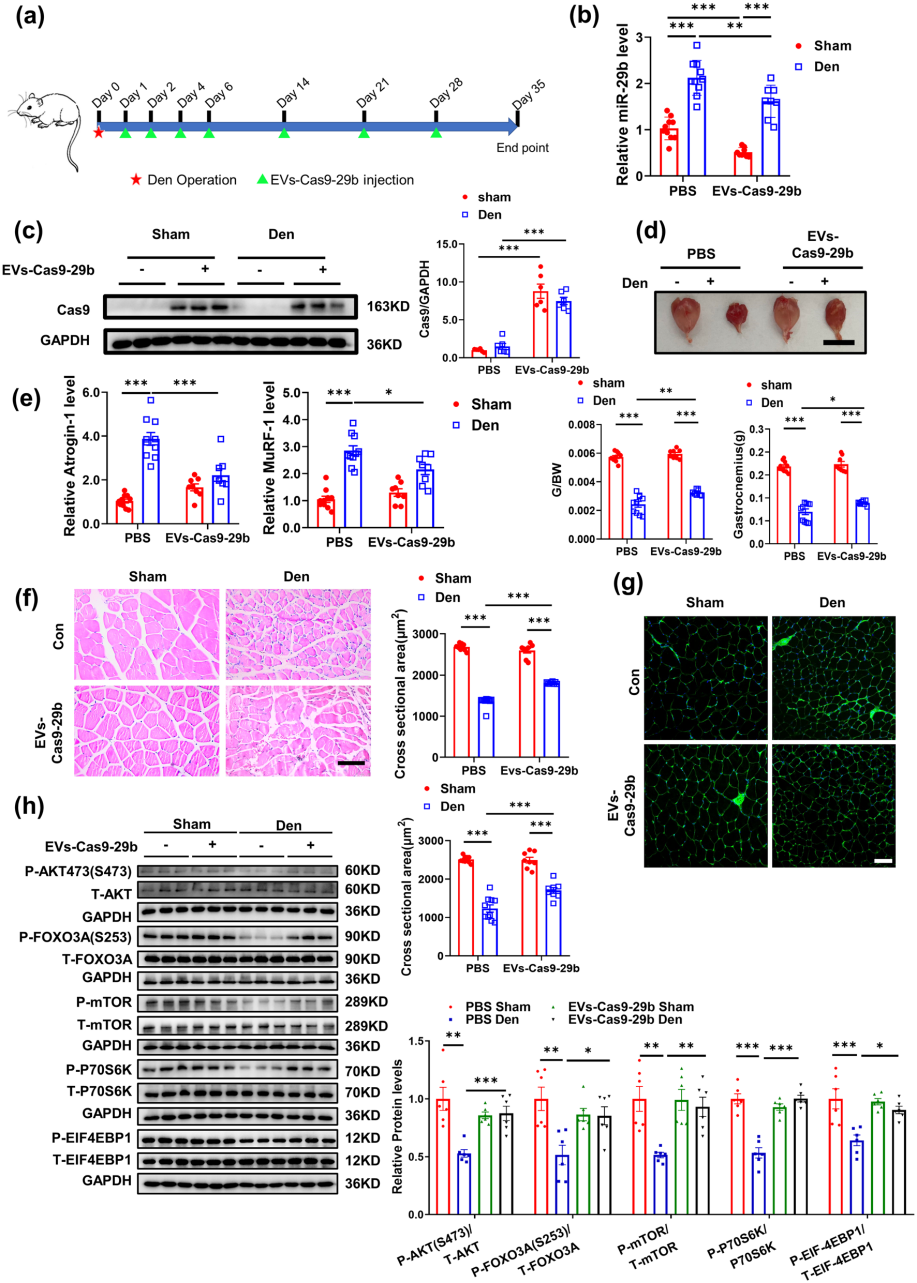
EVs-Cas9-29b therapy attenuate denervation induced muscle atrophy in vivo. **a** Schematic diagram of the experimental design process. **b** qRT-PCR analysis for the expression of miR-29b in gastrocnemius muscle of mice injected with EVs-Cas9-29b in Den-induced muscle atrophy (n = 10,10,8,8). **c** Western blot analysis of the Cas9 protein in gastrocnemius muscle of mice injected with EVs-Cas9-29b in Den-induced muscle atrophy (n = 6). **d** Gastrocnemius muscle morphology, weight and Gastrocnemius weight/body Weight ratio of mice injected with EVs-Cas9-29b in Den-induced muscle atrophy (n = 10,10,8,8). **e** qRT-PCR analysis of Atrogin-1 and MuRF-1 expression in gastrocnemius muscle of mice injected with EVs-Cas9-29b in Den-induced muscle atrophy (n = 10,10,8,8). **f–g** HE staining and WGA staining for myofiber of mice injected with EVs-Cas9-29b in Den-induced muscle atrophy (HE:n = 9,10,8,8; scale bar:100 μm; WGA: n = 10,10,8,8; scale bar:100 μm). **h.** Western blot analysis for AKT/FOXO3A/mTOR pathway (AKT, FOXO3A, mTOR, P70S6K, 4EBP1) in gastrocnemius muscle of mice injected with EVs-Cas9-29b in Den-induced muscle atrophy (n = 6 per group). Data were presented as mean ± SD. Statistical significance was determined by Two-way ANOVA with Tukey test (**b**–**h**). *P < 0.05; **P < 0.01 and ***P < 0.001

We further determined the therapeutic effects of EVs-Cas9-29b on denervation (Den) -induced muscle atrophy. As previously detected in the IMO-induced muscle atrophy model, the mice were subjected to denervation surgery, followed by the injection of EVs-Cas9-29b into the gastrocnemius muscle for 5 weeks of therapy (Fig. 6a). After confirming that EVs-Cas9-29b was indeed delivered and worked efficiently in skeletal muscles (Fig. 6b, c and Additional file 1: Fig. S4a), a similar approach was undertaken in the IMO-induced muscle atrophy model to detect EV delivery and efficacy. EVs-Cas9-29b could treat Den-induced muscle atrophy by increasing the muscle weight and cross-sectional area of myofibers, suppressing Atrogin-1 and MuRF-1 expression and reactivating the AKT-FOXO3A-mTOR signaling pathway (Fig. 6d–h). Besides, EVs-Cas9-29b could restore the expression of IGF-1 and PI3K(p85α) (Additional file 1: Fig. S4b). Furthermore, EVs-Cas9-29b therapy does not cause an inflammatory response in the muscles of the denervated mice (Additional file 1: Fig. S4c). Taken together, our study suggests that EVs-Cas9-29b could be a promising therapy for Den-induced muscle atrophy.

## Discussion

In this study, we provide a muscle atrophy therapy method by using an engineered extracellular vesicle delivery system, that carries the miR-29b editing system mediated by CRISPR/Cas9 (EVs-Cas9-29b).

miRNAs have been reported to play a crucial role in various biological processes and in many disease phenotypes. miRNAs were thought to be likely therapeutic targets due to their small size and powerful regulation. Thus, the investigation of miRNA therapy for diseases has garnered increasing interest from researchers [26]. Unfortunately, until now, no miRNA therapy has been used in clinical practice, even though some have entered phase I or phase II clinical trials. There are some limitations to miRNA therapy. Off-target effects and toxicity are critical obstacles in miRNA drug development [26]. To increase stability, chemically modified oligonucleotides are being used in clinical trials and preclinical stages. Synthetic double-stranded small RNA molecules have been used to restrict the corresponding miRNA using methods such as antisense oligonucleotides (ASOs), modification of locked nucleic acids (LNAs), and 2ʹ-O-methoxyethyl modified antagomirs [27]. Due to the induction of an abnormal immune response, the clinical trials for MRX34 have been halted [28]. Thus, newer strategies need to be conceived to avoid such complications. The CRISPR/Cas9 system is not only a precise genome modification method and a promising tool for therapeutic applications but could act as a powerful inhibitor of miRNA with a lasting effect. Therefore, we chose to develop a novel CRISPR-based genome editing method against miR-29b for muscle atrophy therapy. CRISPR/Cas9 system was previously delivered using lentivirus and adeno-associated viruses (AAV) for in vivo gene therapy [10]. Due to the limitations of viral genomic DNA packaging capacity (5 KB) and safety during conventional viral delivery, further clinical translational research is required so as to develop a more appropriate and safer transport system.

With multiple advantages such as overcoming the natural barriers, stability in circulation, low immunogenicity, high packaging capacity, and high engineerability, EVs are considered as promising vehicles for drug delivery [21, 29]. Importantly, EVs have also been explored as delivery vehicles for Cas9 RNPs for genome editing [30–32]. NanoMEDIC [14], GEDEX (Genome editing with designed extracellular vesicles) [19], ARMMs [33], Nanoblades [34], VEsiCas [18], Gesicles [17], and aptamer/ABP system [20] have all been reported to transport the CRISPR/Cas9 system for gene editing. NanoMEDIC is an all-in-one EV delivery system, with a fused FKBP12 with lentiviral Gag protein, and fused FRB with SpCas9 protein. The FKBP12 and FRB interaction is induced by AP21697, thus bringing Cas9 protein to Gag and is then packaged into EVs. A long RNA, triggered by HIV Ψ packaging signal, undergoes self-cleavage by ribozymes and releases the sgRNA when being loaded into EVs. NanoMEDIC reduces off-target activity and improves the on- to off-target ratio of DNA plasmid. This weighs favorably for the clinical application of the NanoMEDIC system. The miR-29b editing system (EVs-Cas9-29b), delivered by the NanoMEDIC system, could repress the expression of miR-29b by introducing mutagenesis in pri-miR-29b-1 and − 2. This shows that the editing system could be successfully transported into the skeletal muscle cells. Importantly, as the EVs-Cas9-29b were derived from myoblasts, they have low immunogenicity during the skeletal muscle therapy. We also discovered that inflammation-related genes (TNF-, IL-6, and IL-1b) showed no changes even after 5 weeks of EVs-Cas9-29b therapy in IMO- and Den-induced muscle atrophy which is coincidentally the endpoint of the treatments. Derived from C2C12 myoblasts, the components from myoblast, such as DNA, protein, or RNA may package in EVs-Cas9-29b. Fortunately, the components from myoblast are usually not toxic for the skeletal muscles. Thus, EVs-Cas9-29b was suitable for skeletal muscle therapy. Still, future studies will need to focus on removing any redundant components of EVs-Cas9-29b.

Compared to viral delivery, engineered EVs lacking viral nucleic acid genome have made the “carrier” safer without their insertion in the genome of the recipient cell (Additional file 1: Fig. S2). Packing Cas9 protein, rather than DNA plasmid expression vectors, lets the system work faster, which makes this a more effective therapeutic choice for early treatment. Using this system, an effective therapy has been demonstrated for established muscle atrophy models induced by immobilization and denervation, which makes this system more suitable for clinical application. Comparing the aspect of therapeutic efficacy, while AAV8-delivered CRISPR/SaCas9 therapy increased the muscle weight by 10.8% in IMO-induced muscle atrophy as shown in our previous study, EVs-Cas9-29b therapy improved the muscle weight further by 22.5% as observed in this study. Additionally, Cas9 protein can be degraded in the recipient cells, unlike AAV-mediated Cas9 expression that persists for a long time. Short-lived Cas9 protein could reduce the probability of off-target mutagenesis.

Although the Cas9 protein was only enriched in the gastrocnemius muscle in our therapy experiments via intramuscular injection, targeted delivery of EVs-Cas9-29b should be further developed. It is exciting to model targeted therapy using surface engineering of EVs which could be an effective method to confer cell-type targeting specificity. By adding a guiding protein or targeting polypeptide to the membrane protein of EVs, there have been reports of specific tissue or organ targeting EVs that were successfully used in pre-clinical stages. For example, rabies virus glycoprotein (RVG) peptide has been used to develop neuro-specific EV [35]. miR-124-loaded RVG EVs could be transferred to ischemic regions of the cortex and promote neurogenesis by intravenous injection [36]. Engineered chondrocyte affinity peptide EVs could carry miR-140 to chondrocytes in the joints and ameliorate osteoarthritis [37]. After extensive screening, certain skeletal muscle targeting peptides could be identified and ligated onto the extra- membrane of EVs to improve the targeted therapy against skeletal muscular diseases.

There are a few limitations to this study. As a proof of concept for clinical application, the comprehensive systemic effects or unwelcome side effects should have been taken into consideration. These aspects need to be taken into account in future studies to improve the clarity, purity, efficiency, efficacy and specificity during such targeted-therapeutic EVs production.

## Conclusions

In conclusion, we successfully constructed an engineered extracellular vesicle delivery system for miR-29b gene-editing that could be used as an effective therapeutic strategy against muscle atrophy.

## Methods

### Plasmids

Plasmids required for packaging EVs were obtained from Addgene, including pHLS-EF1a-FKBP12-Gag (Addgene ID: 138,476); pHLS-EF1a-FRB-SpCas9-A (Addgene ID: 138,477); pcDNA1-Tat (Addgene ID: 138,478); pVSV-G (Addgene ID: 138,479). The construction method of vector plasmid carrying sgRNA is as follows:

Step 1 - Clone sgRNA into pENTR-AmCyan.

To clone sgRNA (targeting sequence 5′-CCTAAAACACTGATTTCAAA-3′ site) into pENTR-AmCyan (Addgene ID: 138,481), the fragment containing sgRNA 5′-CGGCCGACCGAATTCGCGGCCTTTAGGCTGATGAGTCCGTGAGGACGAAACGAGTAAGCTCGTCCCTAAAACACTGATTTCAAAGTTTTAGAGCTATGCTGGAAACAGCATAGCAAGTTAAAATAAGGCTAGTCCGTTATCAACTTGAAAAAGTGGCACCGAGTCGGTGCTTTTTTTTTGGCCGGCATGGTCCCAGCCTCCTCGCTGGCGCCGGCTGGGCAACATGCTTCGGCATGGCGAATGGGACGGCCGCTCTAGAACTCGGCCG-3′ was synthesized by GENEWIZ (Suzhou, China). pENTR-AmCyan is digested into linear by restriction enzyme *EgaI* (NEB, MA, USA, R3505), and the synthesized fragment is cloned into the vector using ClonExpress MultiS one-step cloning kit (Vazyme, Nanjing, China, C113-01).

Step 2 - Clone sgRNA into PL-5 L-GW-A.

Gateway™ LR Clonase™ II Enzyme mix (Thermo Fisher Scientific, MA, USA, 11,791,020) was performed with PL-5 L-GW-A-sgRNA and PL-5 L-GW-A to obtain PL-5 L-RGR (miR-29b)-AmCyan-A.

### Cell culture and treatment

C2C12 cells (mouse skeletal myoblasts) were obtained from ATCC and cultured in Dulbecco’s modified Eagle’s medium (DMEM, Corning, NYC, USA, 10-013-CV) with 10% Fetal bovine serum (Biological Industries, Beit HaEmek, Israel) and 1% Penicillin-Streptomycin (KeyGEN, Nanjing, China, KGY0023) at 37 ℃ supplemented with 5% CO_2_. To differentiate into myotubes, C2C12 myoblasts cells were cultured in a differentiation medium (DMEM containing 2% horse serum and 1% penicillin and streptomycin) and the entire differentiation process lasted about 4 days. EV-depleted fetal bovine serum (FBS) was obtained after overnight centrifugation at 100,000 *g* at 4 °C (Beckman Coulter, Avanti JXN-30). For screening the optimum concentration, EVs were added to the medium for 24 h at different doses. For determining the function of EVs in muscle atrophy in vitro, EVs were added to the medium at a dose of 1 × 10^10^ particles/mL for 24 h, then muscle atrophy was induced. Dexamethasone (Dex, Sigma, MO, USA, D4902), TNF-α (PeproTech, NJ, USA, 315-01 A), and Ang II (Sigma, MO, USA, A9525) were used to induce muscle atrophy at the cellular level according to previously reported methods [4].

### EVs production

C2C12 cells were seeded onto a 10 cm culture dish at the density of 4 million/dish. When the cells proliferated to 80% density, pHLS-EF1a-FKBP12-Gag (10 µg), pHLS-EF1a-FRB-SpCas9-A (10 µg), pcDNA1-Tat (2 µg), pVSV-G (5 µg), PL-5 L-29b-AmCyan-A (10 µg) were transfected into the cells using the transfection reagent PEI Max (1 µg/µL; Polyethylenimine Linear MW40000, Kingmorn, Shanghai, China KE1098). The ratio of plasmid and transfection reagent was 1:3 (m/m). 12 h after transfection, medium was changed using exosome-free FBS, and the cell culture supernatant was collected 48 h after the medium change.

### Isolation and purification of EVs

First, the collected medium was centrifuged at 300⋅g for 10 min at 4 °C to remove all living cells. Subsequently, the supernatant was transferred to a new centrifuge tube, and the dead cells were removed by centrifugation at 2,000 *g* for 10 min at 4 °C. The supernatant was then transferred to a new centrifuge tube, centrifuged at 10,000⋅*g* for 30 min at 4 °C to remove cell debris, and finally the supernatant was transferred onto an ultracentrifuge tube, and centrifuged at 4 °C at 100,000 *g* for 70 min to obtain EVs. An appropriate amount of PBS was used to dissolve the pellet.

### Fluorescent nanoparticle tracking analysis (FNTA)

In order to exclude non-membrane-structured nanoparticles and to detect the content of exosomes in nanoparticles more accurately, extracted EVs (1.4 × 10^10^ particles) were incubated with 10 µg/mL DiO (Beyotime Biotechnology, Shanghai, China, C1038) at 37 ℃ for 30 min and then centrifuged at 100,000×*g* for 70 min to remove excess dye, and fluorescently labeled EVs were resuspended in 2 ml sterile PBS. To calibrate the accuracy of the ZetaView Nanoparticle Tracking Analyzer (Particle Metrix) for measuring fluorescent EVs, fluorescent PS beads YG-488 (Particle Metrix, 120 − 0102) were diluted 250,000 times using sterile H_2_O without nanoparticles, and were injected into the instrument and calibrated. Then, the fluorescently labeled EVs were diluted 2,500 times using sterile PBS without nanoparticles, and the fluorescently labeled EVs were injected into the instrument using a syringe to set the parameters according to the instrument’s operation manual, by tracking the Brownian motion of the exosomes and combining the Stokes-Einstein equation to calculate the number of particles of fluorescently labeled EVs and the size of particle diameter. The EV measurements were repeated three times for each sample.

### Transmission electron microscopy (TEM)

For TEM analyses to identify EVs, the purified exosomes were resuspended in PBS without nanoparticles, and 20 µl of EVs were mixed with an equal volume of 4% PFA. The fixed EVs were further diluted 20-fold with 2% PFA, 5 µl of this was put on the copper mesh, was let to stand for 1 min, then filter paper was used to absorb excess liquid. After being washed with double distilled water for three times and the copper mesh was left to dry naturally, and the EVs were then observed using electron microscope. The images were captured under a LVEM5 transmission electron microscope (Delong America, Montreal, QC, Canada).

### Validation of EVs uptake

Extracted EVs were incubated with 2 µg/mL DiD (Beyotime Biotechnology, Shanghai, China, C1039) at 37 ℃ for 30 min and then centrifuged at 100,000 ×*g* for 70 min to remove excess dye. C2C12 cells were seeded onto 12-well plates at a concentration of 40,000 cells /ml. After 12 h, DiD-labeled EVs were added to the cells. After an incubation of 4 h, the cell supernatant was removed, washed three times with PBS, and digested with trypsin. Then, the cells were resuspended in PBS, their fluorescence intensity was detected using a flow cytometry (CytoFlex, Beckman, USA). To detect cellular EV uptake with confocal microscopy, C2C12 cells were seeded onto µ-Slide 8 well glass plates (Ibidi, Gräfelfing, Germany) at a density of 10,000 cells/ml, and DiD-labeled EVs were added to the cells after 12 h. After an incubation of 4 h, cells were washed 3 times with PBS, and then fixed with 4% PFA for 15 min. The nuclei were then stained with DAPI (KeyGEN, China, KGA215) and imaging of the uptake of fluorescently DiD-labeled EVs by C2C12 was detected using confocal microscopy (FV3000, Olympus, Japan).

### Quantitative real-time polymerase chain reactions (qRT-PCR)

Total RNA in cells or tissues was extracted using RNA isolater Total RNA Extraction Reagent (Vazyme, Nanjing, China, R401-01). Subsequently, the RNA reverse transcription experiment was performed using the SuperScript First-Strand Synthesis System for RT-PCR (Thermo Fisher Scientific, MA, USA, 11,904,018) to synthesize cDNA. Real-time fluorescent quantitative PCR experiments were performed using the SYBR Green PCR kit (Takara, Shiga, Japan RR820A). The relative mRNA levels were measured using the 2^−ΔΔCq^ method and 18 S rRNA was used as the internal control. For miRNA, 5 S rRNA was used as the internal control and the bulge loop miRNA qPCR primer set (RiboBio, Guangzhou, China) was used. The primers used are as follows:

mmu-18S rRNA-Forward: 5′-TCAAGAACGAAAGTCGGAGG-3′;

mmu-18S rRNA -Reverse: 5′-GGACATCTAAGGGCATCAC-3′;

mmu-Atrogin-1- Forward: 5′-CAGCTTCGTGAGCGACCTC-3′;

mmu-Atrogin-1- Reverse: 5′-GGCAGTCGAGAAGTCCAGTC-3′;

mmu-MuRF-1- Forward: 5’-GTGTGAGGTGCCTACTTGCTC-3’;

mmu-MuRF-1- Reverse: 5′-GCTCAGTCTTCTGTCCTTGGA-3′;

mmu-IL-1β- Forward: 5′-GCAACTGTTCCTGAACTCAACT-3′;

mmu-IL-1β- Reverse: 5′-ATCTTTTGGGGTCCGTCAACT-3′;

mmu-IL-6- Forward: 5′-TAGTCCTTCCTACCCCAATTTCC-3′;

mmu-IL-6- Reverse: 5′-TTGGTCCTTAGCCACTCCTTC-3′;

mmu-TNF-α- Forward: 5′-AGGCACTCCCCCAAAAGATG-3′;

mmu-TNF-α- Reverse: 5′-CCACTTGGTGGTTTGTGAGTG-3′.

### T7 endonuclease I (T7EI) assay

The genomic DNA of the tissue or cells treated with EVs were used as template, and the fragment encoding miR-29b was amplified by PCR using specific primers: miR-29b-1 Forward: 5′-GCTGCACCGTGAATGTGTAA-3′, miR-29b-1 Reverse: 5′-AGGTCTTCATCCGAGCATGG-3′; miR-29b-2 Forward: 5′-TGTACATATGTTGAATGGATTTGGT-3′, miR-29b-2 Reverse: 5′-TGCTGCAACCAGGACTGAAT-3′. KOD Plus Neo (Toyobo, Osaka, Japan, KOD-401) was used for PCR. The purified PCR product was denatured and annealed in NEB buffer 2 (NEB, MA, USA, B7002S) in a total volume of 20µL. The denaturation and annealing steps are as follows: 95 ℃, 5 min; 95–75 ℃, − 0.1 ℃/cycle, 200 times; 75–15℃, − 0.1℃/cycle, 600 times; hold at 4 ℃. 1U of T7EN1 enzyme (NEB, MA, USA, M0302S) was added to the PCR product and digested at 37 °C for 1 h, followed by separation of the digested product using 2% agarose, stained with GelRED, and images were captured by ChemiDoc XRS (Bio-Rad, PA, USA).

### Mouse models

Eight-week-old male C57BL/6J mice were purchased from Charles River (Beijing, China) and maintained in the SPF laboratory animal facility of Shanghai University (Shanghai, China). All procedures with animals were performed in accordance with the guidelines on the use and care of laboratory animals for biomedical research published by the National Institutes of Health (No. 85 − 23, revised 1996), and the experimental protocol was reviewed and approved by the ethical committee of Shanghai University. A detailed method of the mouse muscle atrophy models has been previously described in our work [4]. Briefly, for the mouse muscle atrophy treatment experiment, the mouse sciatic nerve was cut to make a denervation muscle atrophy model and the sham mice were generated by the same process but without cutting off the sciatic nerve. The hind limbs of the experimental mice group were fixed by a screw (0.4 × 8 mm) to construct an immobilization muscle atrophy model and while the sham mice were not fixed. For EVs-Cas9-29b therapy, the mice received an intramuscular injection of EVs at 1, 2, 4, 6, 14, 21, and 28 days after surgery at the dose of 8.5 × 10^10^ particles/mice. The mice were sacrificed on day 35. Gastrocnemius was harvested, muscle weight and body weight were measured. Muscle specimens were either immediately snap-frozen using liquid nitrogen and stored at -80 °C for further analyses, or embedded for histological analyses.

### Western blot

PI buffer containing PMSF (KeyGEN, Nanjing, China, KGP701) was used to prepare protein lysates for cell and tissue samples. The BCA protein assay kit (Thermo Fisher Scientific, MA, USA, 23,225) was used to determine the concentration of protein samples. The protein samples were separated using SDS-PAGE gel, and the proteins were then transferred onto a PVDF membrane, which was then blocked and incubated with the primary antibody and the secondary antibody consecutively, and then visualized using the High-sig ECL Western Blotting Substrate (Tanon, Shanghai, China, 180–501). GAPDH was used as the loading control. Primary antibodies used in this study were as follows: P-AKT (S473) (1:1000, Cell Signaling Technology, MA, USA, 4060 S), AKT (1:1000, Proteintech, Wuhan, China, 10176-2-AP), P-FOXO3A (S253) (1:1000, Cell Signaling Technology, MA, USA, 9466 S), FOXO3A (1:1000, Abclonal Technology, Wuhan, China, A9270), P-mTOR (1:1000, Cell Signaling Technology, MA, USA), mTOR (1:1000, Cell Signaling Technology, MA, USA, 2972 S), P-P70S6K (1:1000, Cell Signaling Technology, MA, USA, 9204 S), P70S6K (1:1000, Cell Signaling Technology, MA, USA, 9202 S), P-4EBP1 (1:1000, Abclonal Technology, Wuhan, China, AP0030), 4EBP1 (1:1000, Abclonal Technology, Wuhan, China, A19045), Cas9 (1:1000, abcam, Cambridge, England, ab191468), VSV-G (1:1000, abcam, Cambridge, England, ab183497), CD9 (1:1000, Santa Cruz, TX, USA, sc-13,118), CD63 (1:1000, Santa Cruz, TX, USA, sc-5275), PI3 Kinase P85-α (1:500, Proteintech, Wuhan, China, 11748-1-AP), IGF-1 (1:500, Abclonal Technology, Wuhan, China, A12305), TSG-101 (1:500, Abclonal Technology, Wuhan, China, A1692), TOM-20 (1:1000, Proteintech, Wuhan, China, 11802-1-AP), Calnexin (1:500, Abclonal Technology, Wuhan, China, A15631), APOA1 (1:500, Abclonal Technology, Wuhan, China, A14211) and GAPDH (1:10000, Bioworld Technology, Nanjing, China, AP0063).

### Immunofluorescence staining

Myotubes were fixed with 4% paraformaldehyde for 20 min at room temperature. After permeabilizing with 0.5% Triton X-100, the myotubes were blocked with 5% bovine serum albumin (BSA) for 2 h at room temperature. They were then incubated with primary antibody MF-20 (1:100, DSHB, IA, USA, AB-2,147,781) overnight at 4 °C. The next day, the samples were incubated with their corresponding secondary fluorescence conjugated antibodies, Alexa Fluor® 488 AffiniPure Goat Anti-Mouse IgG (H + L) (1:200, Jackson ImmunoResearch Laboratories, PA, USA, 115-545-003) at room temperature for 2 h while being protected from light. After washing the samples with PBS, nuclei were stained for DAPI (1:2000, KeyGen, Nanjing China, KGA215) and imaged. Leica fluorescence microscope (Wetzlar, Germany, DM i8) with a 20× objective lens was used to take fluorescence images. The diameter of the myotubes were analyzed using Image J software (NIH, USA) and a minimum of 40 myotubes were imaged for each group.

### Detection and statistics of muscle fiber cross-sectional area

H&E staining and wheat germ agglutinin (WGA) staining were used to detect the cross-sectional area of mouse gastrocnemius muscle fibers, which has been described in detail in our previous study [10].

For H&E staining, each slide was imaged using a 20× magnification on a Leica microscope (Wetzlar, Germany, DM3000) to take 20-40 fields of view per sample. The area of the myofibers was analyzed using Image J software (NIH, USA), with a minimum of 400 fibers per mouse being analyzed.

WGA lectin staining was performed as it is a fast, reliable and an inexpensive method used for skeletal muscle fiber, compared to other antibody-based techniques[38]. The frozen tissue sections were stained with WGA (1:200, Sigma, MO, USA), and photographed with Carl Zeiss fluorescence microscope (Oberkochen, Germany, Axio Imager M2) at 20× magnification, and the area of the myofibers was analyzed with Image J software with a minimum of 400 fibers per mouse being counted.

### Statistical analysis

Data are represented as mean ± SD. An unpaired, two-tailed Student’s t test was used for comparisons between the two groups. Two-way ANOVA with Tukey test was performed to compare multiple groups. All analyses were performed using GraphPad Prism 8.0. Differences with p < 0.05, were considered significant.

## Supplementary Information

**Additional file 1: Figure S1.** EVs-Cas9-miR-29b does not affect the expression of miR-29b-5p-1/2 **Figure S2.** EVs-Cas9-29b lacks viral nucleic acid genome **Figure S3.** Enrichment of Cas9 protein, the expression of IGF-1, PI3K(p85α), and the expression of inflammation-related genes in mice therapy with EVs-Cas9-29b in immobilization induced muscle atrophy **Figure S4.** Enrichment of Cas9 protein, the expression of IGF-1, PI3K(p85α), and the expression of inflammation-related genes in mice therapy with EVs-Cas9-29b in denervation induced muscle atrophy.

## Data Availability

The authors declare that all data supporting the findings of this study are available within the paper and Additional file. The materials used in this study is available from the corresponding author upon reasonable requests.

## Abbreviations

EVs: Extracellular vesicles
Dex: Dexamethasone
AngII: Angiotensin II
TNF-α: Tumor necrosis factor alpha
CRISPR: Clustered regularly interspaced short palindromic repeat
AAV: Adeno-associated virus
FNTA: Fluorescent nanoparticle tracking analysis
TEM: Transmission electron microscopic assays
IMO: Immobilization
Den: Denervation
MuRF-1: Muscle-specific RING-finger 1
atrogin-1/ MAFbx: muscle atrophy F-box
T7EI: T7 Endonuclease I
