# Supplementary material for: Delivery of engineered extracellular vesicles with miR-29b editing system for muscle atrophy therapy
Source: J Nanobiotechnology. 2022 Jun 27;20:304. doi: 10.1186/s12951-022-01508-4 (PMC9235146; doi:10.1186/s12951-022-01508-4)
Supplement: Supplementary file 1 — Additional file 1: Figure S1. EVs-Cas9-miR-29b does not affect the expression of miR-29b-5p-1/2 Figure S2. EVs-Cas9-29b lacks viral nucleic acid genome Figure S3. Enrichment of Cas9 protein, the expression of IGF-1, PI3K(p85α), and the expression of inflammation-related genes in mice therapy with EVs-Cas9-29b in immobilization induced muscle atrophy Figure S4. Enrichment of Cas9 protein, the expression of IGF-1, PI3K(p85α), and the expression of inflammation-related genes in mice therapy with EVs-Cas9-29b in denervation induced muscle atrophy. [file 12951_2022_1508_MOESM1_ESM.docx]

**Additional file 1**

**Delivery of engineered extracellular vesicles** **with miR-29b editing system for muscle atrophy therapy**

Rui Chen^1,2,#^, Weilin Yuan^1,2,#^, Yongjun Zheng^3,#^, Xiaolan Zhu^1,2^, Bing Jin^1,2^, Tingting Yang^1,2^, Yuwei Yan^1,2^, Wanru Xu^1,2^, Hongjian Chen^1,2^, Juan Gao^1,2^, Guoping Li^4^, Priyanka Gokulnath^4^, Gururaja Vulugundam^5^, Jin Li^1,2,*^, Junjie Xiao^1,2,*^

^1^Institute of Geriatrics (Shanghai University), Affiliated Nantong Hospital of Shanghai University (The Sixth People’s Hospital of Nantong), School of Medicine, Shanghai University, Nantong 226011, China

^2^Cardiac Regeneration and Ageing Lab, Institute of Cardiovascular Sciences, Shanghai Engineering Research Center of Organ Repair, School of Life Science, Shanghai University, Shanghai 200444, China

^3^Division of Pain Management, Huadong Hospital Affiliated to Fudan University, Shanghai 200040, China

^4^Cardiovascular Division of the Massachusetts General Hospital and Harvard Medical School, Boston, MA 02114, USA.

^5^Institute of Biochemistry and Cellular Biology, National Research Council of Italy, Napoli 80131, Italy

#These authors contributed equally to this work.

*Correspondence should be addressed to

Dr. Jin Li

Cardiac Regeneration and Ageing lab, Institute of Cardiovascular Sciences, Shanghai Engineering Research Center of Organ Repair, School of Life Science, Shanghai University, 333 Nan Chen Road, Shanghai 200444, China

Tel: +86-21-66138131; Fax: +86-21-66138131

E-mail: sszxlijin@163.com

Dr. Junjie Xiao

Cardiac Regeneration and Ageing lab, Institute of Cardiovascular Sciences, Shanghai Engineering Research Center of Organ Repair, School of Life Science, Shanghai University, 333 Nan Chen Road, Shanghai 200444, China

Tel: +86-21-66138131; Fax: +86-21-66138131

E-mail: [junjiexiao@shu.edu.cn](mailto:junjiexiao@shu.edu.cn)

**Additional Figures**


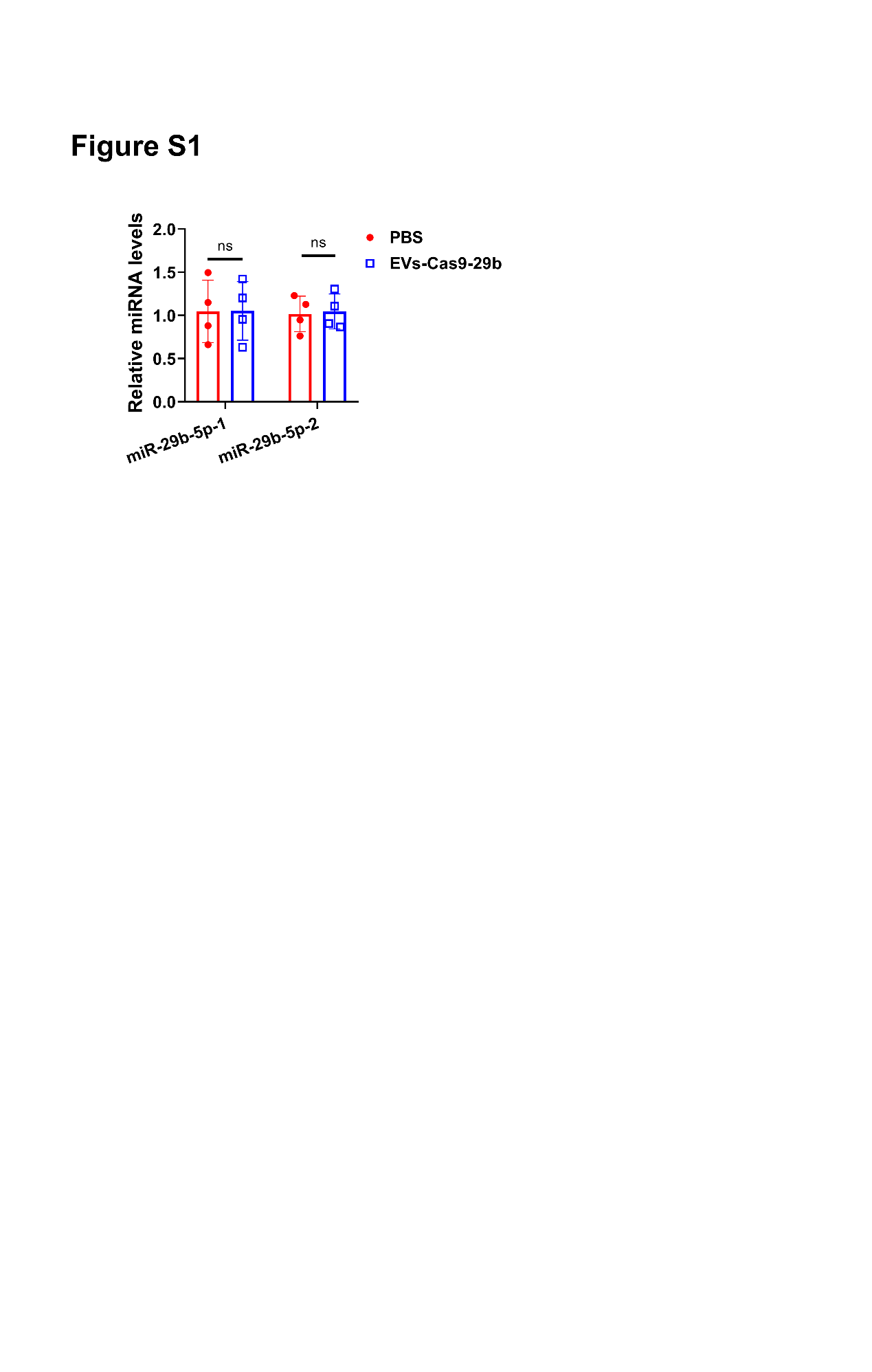


**Figure S1.** **EVs-Cas9-miR-29b does not affect the expression of miR-29b-5p-1/2.**

Real-time PCR analysis of the expression of miR-29b-5p-1 and miR-29b-5p-2 in C2C12 myotubes after treatment with EVs-Cas9-29b (n=4 per group). ns, no significance.

**
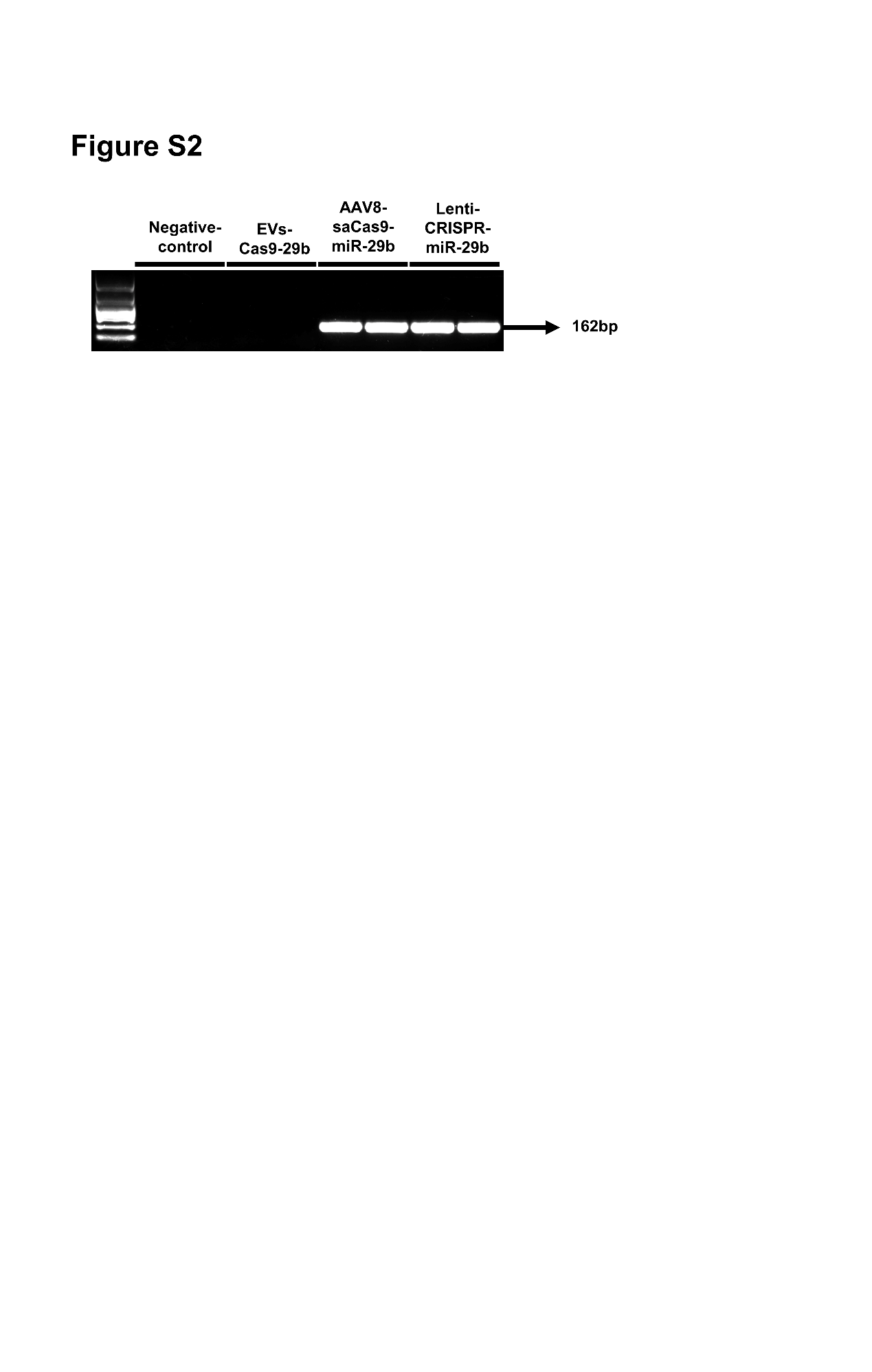
**

**Figure S2.** **EVs-Cas9-29b lacks the viral nucleic acid genome.**

DNA electrophoresis using agarose gel for detection of viral nucleic acid genome in EVs-Cas9-29b, AAV8-SaCRISPR-miR-29b and Lenti-CRISPR-miR-29b.


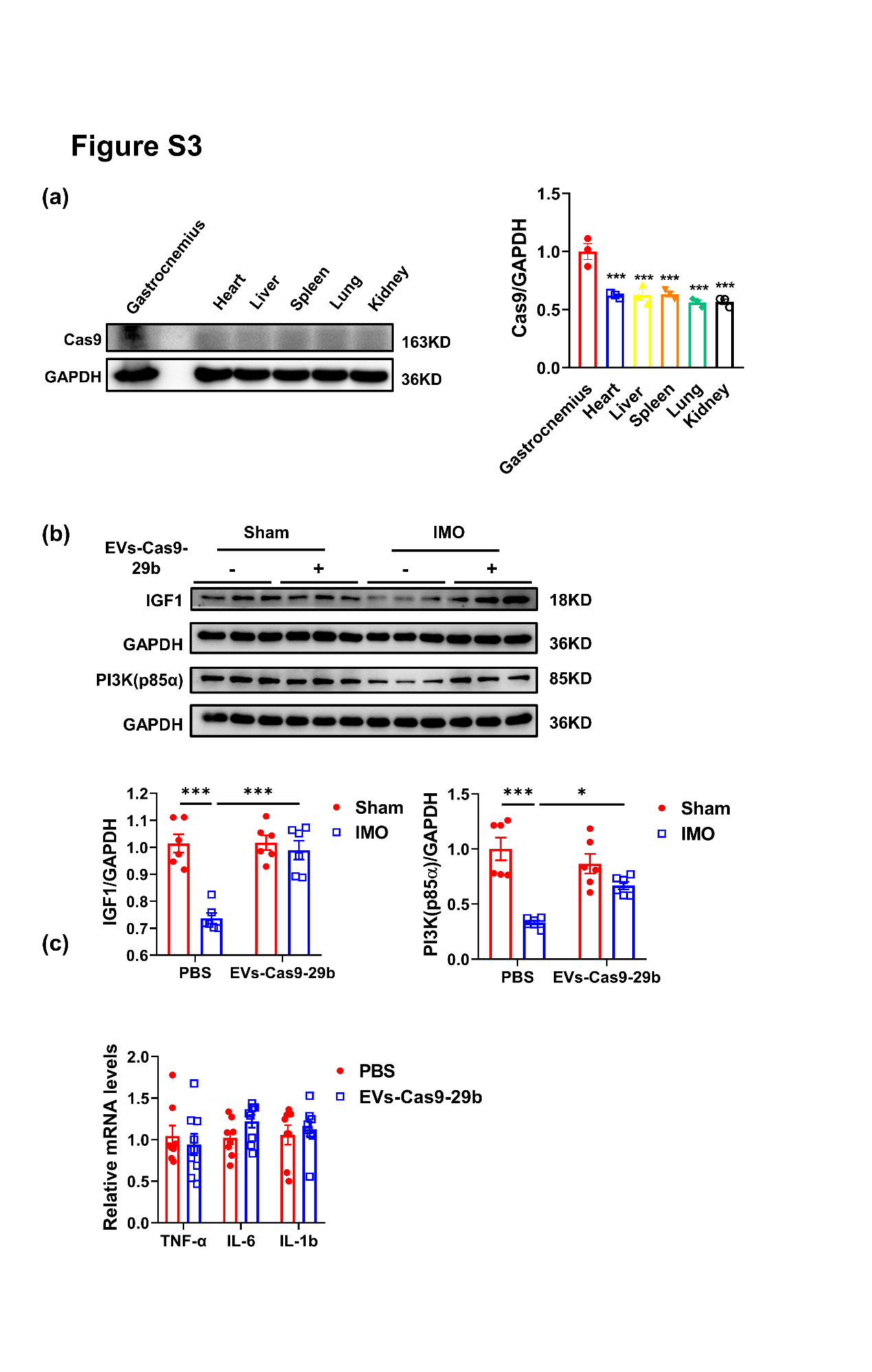


**Figure S3. Enrichment of Cas9 protein, the expression of** **IGF-1, PI3K(p85α), and inflammation-related genes in mice treated with EVs-Cas9-29b in** **immobilization induced muscle atrophy.**

**a.** Western blot analysis of the Cas9 protein in gastrocnemius muscle, Heart, Liver, Spleen, Lung, and Kidney of mice injected with EVs-Cas9-29b in immobilization (IMO)-induced muscle atrophy (n=3). **b.** Western blot analysis for IGF-1, PI3K(p85α) in gastrocnemius muscle of mice injected with EVs-Cas9-29b in IMO-induced muscle atrophy (n=6 per group). **c.** qRT-PCR analysis for the expression of TNF-α, IL-6 and IL-1b in gastrocnemius muscle of mice injected with EVs-Cas9-29b in IMO-induced muscle atrophy(n=8,9). Data were presented as mean±SD. Statistical significance was determined by Student t test (a,c) and Two-way ANOVA with Tukey test (b). *, P<0.05; ***, P<0.001.

**
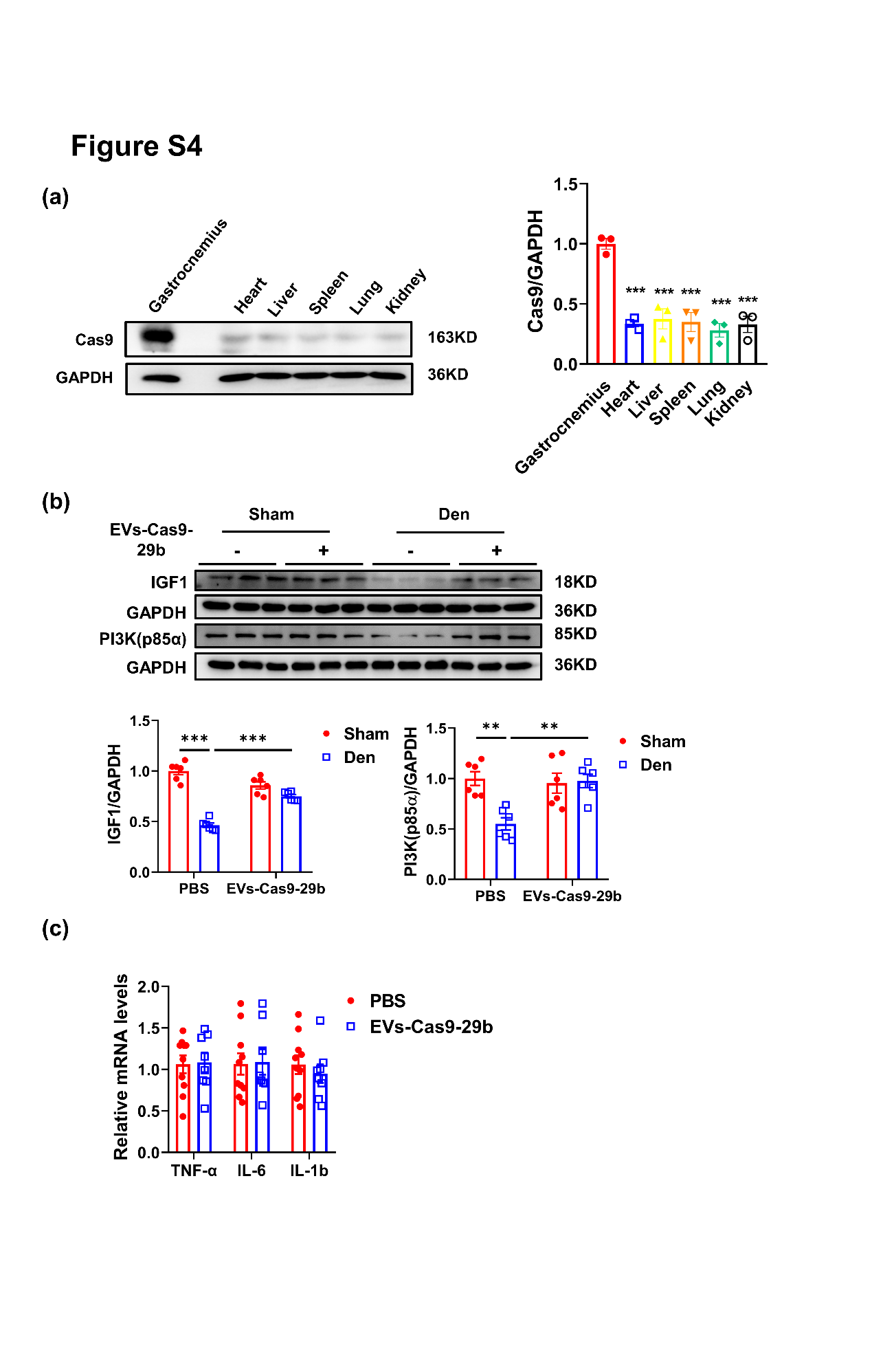
**

**Figure S4. Enrichment of Cas9 protein, expression of IGF-1, PI3K(p85α), and i****nflammation-related genes in mice treated with EVs-Cas9-29b in** **denervation induced muscle atrophy.**

**a.** Western blot analysis of Cas9 protein in gastrocnemius muscle, Heart, Liver, Spleen, Lung, and Kidney of mice injected with EVs-Cas9-29b in denervation (Den)-induced muscle atrophy (n=3). **b.** Western blot analysis of IGF-1, PI3K(p85α) in gastrocnemius muscle of mice injected with EVs-Cas9-29b in Den-induced muscle atrophy (n=6 per group). **c.** qRT-PCR analysis for the expression of TNF-α, IL-6 and IL-1b in gastrocnemius muscle of mice injected with EVs-Cas9-29b in Den-induced muscle atrophy(n=10,8). Data were presented as mean±SD. Statistical significance was determined by Student t-test (a,c) and Two-way ANOVA with Tukey test (b). **, P<0.01; ***, P<0.001.
